# Detection and genetic characterization of canine astroviruses in pet dogs in Guangxi, China

**DOI:** 10.1186/s12985-017-0823-4

**Published:** 2017-08-17

**Authors:** Huabo Zhou, Lin Liu, Ruikai Li, Yifeng Qin, Qingli Fang, Vinod RMT Balasubramaniam, Guojun Wang, Zuzhang Wei, Kang Ouyang, Weijian Huang, Ying Chen

**Affiliations:** 1 College of Animal Science and Technology, Guangxi University, No.100 Daxue Road, Nanning, 530004 People’s Republic of China; 2 Huabo Pet Hospital, No.1 Anji Road, Nanning, 53004 People’s Republic of China; 3 Jeffrey Cheah School of Medicine and Health Sciences, Monash University Malaysia, Jalan Lagoon Selatan, 47500 Subang Jaya, Selangor Malaysia; 4 Department of Microbiology, Mount Sinai School of Medicine, New York, NY 10029 USA

**Keywords:** Canine astrovirus, Prevalence, Genetic diversity

## Abstract

**Background:**

Astroviruses (AstVs) have been reported to infect and cause gastroenteritis in most animal species. Human AstVs were regarded the causative agent of viral diarrhea in children. In dogs, little is known about the epidemiology and clinical significance of AstV infection.

**Findings:**

In this study, we collected and tested 253 rectal swabs from pet dogs; of which 64 samples (25.3%) tested positive for AstVs with diarrhea and 15 more samples (5.9%) also was identified as AstVs, however without any clinical signs. Phylogenetic analysis of 39 partial ORF1b sequences from these samples revealed that they are similar to AstVs, which can be subdivided into three lineages. Interestingly, out of the 39 isolates sequenced, 16 isolates are shown to be in the *Mamastrovirus* 5/canine astrovirus (CAstV) lineage and the remaining 23 isolates displayed higher similarities with known porcine astrovirus (PoAstV) 5 and 2. Further, analysis of 13 capsid sequences from these isolates showed that they are closely clustered with Chinese or Italy CAstV isolates.

**Conclusions:**

The findings indicate that CAstVs commonly circulate in pet dogs, and our sequencing results have shown the genomic diversity of CAstVs leading to increasing number of clusters.

Astroviruses (AstVs) are non-lipid enveloped, positive-sense single-stranded RNA icosahedral virus, containing three open reading frames (ORFs). ORF1a and ORF1b were located at the 5′ end of the genome, encoding non-structural proteins. ORF2 encoded a capsid protein, which located at the 3′-terminal end [1]. AstVs were first identified by electron microscopy in 1975 in the stools of infants hospitalized with diarrhea [2]. These star-like viruses can be subdivided into two groups; *Mamastroviruses* which generally infects mammals and *Avastroviruse*s which infects avian species. Both of them are globally distributed. On that note, human AstVs is one of the major causative agent of acute gastroenteritis in young, elderly and immunocompromised patients [1]. AstVs also have been associated with extra intestinal diseases, such the encephalitis in human [1] and cattle [3], interstitial nephritis and growth retardation in chick [4], severe hepatitis in duck [5, 6], and shaking syndrome in mink [7]. Recently, AstVs were also discovered in Chinese bats and rodents [8, 9].

Canine astrovirus (CAstV) was first identified in the early 1980s and recently, it was characterized as a distinct *Mamastrovirus* species, which is the causative agent of gastroenteritis in pet dogs [10–12]. Evidently, CAstVs has spread widely in the dog population and produced higher genetic diverse, as shown Martella et al., where a novel CAstV was identified from dogs with gastroenteritis [13]. However, data on the clinical significance or association of astrovirus infection with other infectious diseases are limited. The aim of our study was to understand the prevalence, genetic diversity and evaluate the risk factors of co-infection with other infectious diseases from the samples collected.

A total of 253 fecal swabs of pet dogs; of which 64 with gastroenteritis and 15 without any syndrome were collected from pet hospitals in Guangxi from November 2015 to June 2016. Clinical signs were recorded in detail, including breed, age, gender, clinical signs. Canine Parvovirus (CPV), Canine Distemper Virus (CDV) and Canine Coronavirus (CCoV) were detected by commercial detection kit, not including the identification of bacterial and parasite pathogens in the stools of all of the pet dogs. The samples of fecal swabs diluted with PBS (200 U/ml penicillin, 200 mg/ml streptomycin and 100 μg/ml gentamicin) were vortexed for 1 min. Virus RNA was extracted from diluted rectal swab by using RNAiso Plus (TAKARA, Dalian), following the manufacturer’s instructions. Reverse transcription (RT) was carried out under standard conditions with stem-loop-2-like motif (S2 m) primer (5′-CCCTCGATCCTACTCGG) located in the 3′ non-coding region [14]. The RT-nested PCR methods developed by Chu et al. [8] were applied to amplify the partial RNA dependent-RNA polymerase (ORF1b). From the samples collected, 64/253(25.3%) and 15/253 (5.9%) resulted RT-PCR positive with gastroenteritis and without any illness, respectively (Table 1). This was higher when compared to recent reports 12% [12], 9.7% [15], 2.1% [16], 6% [17], but it was similar with 24.5% [11] and 20.9% [18] in dogs with diarrhea symptoms. Some of the factors which may have contributed to the differences were the various ages of the dogs investigated, areas, feeding environment, dog population selected and maintenance condition of fecal samples and methods for detection. The prevalence of CAstVs without any symptoms suggest that the persistent replication of CAstVs in the gastrointestinal system and due to the lack of envelop protein, it enables them to have exceptional durability to adapt the harsh gastrointestinal tract and the environment as shown by Lizasoain et al. [19]. The age of dogs investigated in this study has a wide range from 13 days to 15 years old, which was divided into four phases; younger pups (≤6 months), young pups (6 months − 2 years old), adult dogs (2–7 years old) and old dogs (≥7 years old). From the total of 79 positive samples analyzed, we found that the younger puppies were more susceptible, their positive infection rate reached up to 18.6%, but only 5.9% and 3.1% detected in young and adult dogs. There was no detection in older dogs (Table 1). The vulnerability of younger puppies towards infection are dependent on its immune system, feeding environment or stress factor. Apart from that, this may be due also to strain-dependent variations or the synergistic infection of other infectious diseases (CPV-2, CCoV and CDV). Based on the clinical manifestation, out of 79 positive samples, the mixed infection of CPV-2 took up to 23.4% (15/64), CCoV, CDV and CPV-2 + CCoV appeared to be only 3.1% (2/64), 10.9% (7/64) and 3.1% (2/64), respectively (Table 1). A variety of infectious agents may cause enteric sign, so the real pathogenic role of CAstV should be identified by the experimental infections to show that it is the primary causative agent of gastroenteritis in dogs.

**Table 1:** Clinical analysis from the 79 pet dogs with CAstVs infection in 253 rectal samples

Ages	Number (% of Astrovirus positive)(n_i_/n)		Number (% other infection positive)(n_1_/n_2_)			
	Gastroenteritis	Asymptomatic	CPV-2	CCoV	CDV	CPV-2 + CCoV
≤6 months	47/253(18.6)	7/253(2.8)	15/64(23.4)	2/64(3.1)	7/64(10.9)	2/64(3.1)
6 months─2 years old	15/253(5.9)	5/253(2.0)	0/64(0)	0/64(0)	0/64(0)	0/64(0)
2–7 years old	2/253(0.8)	3/253(1.2)	0/64(0)	0/64(0)	0/64(0)	0/64(0)
≥7 years old	0/253(0)	0/15(0)	0/64(0)	0/64(0)	0/64(0)	0/64(0)
Total	64/253(25.3)	15/253(5.9)	15/64(23.4)	2/64(3.1)	7/64(10.9)	2/64(3.1)

n_i_/n = positive number of dogs (including gastroenteritis and asymptomatic, respectively) /total number of dogs

n_1_/n_2_ = positive number of dogs (CPV-2, CCoV, CDV or double infection) /AstV positive number of dogs with gastroenteritis

To confirm the specificity of RT-PCR, 39 of 79 positive samples were purified by Gel extraction Kit (OMEGA, USA) and sequenced. The nucleotide sequences of partial ORF1b genes and partial ORF2 were analyzed and aligned with the Megalign and Seqman program (DNASTAR, Madison, USA). Phylogenetic trees were generated by applying the neighbor-joining method of the Clustal W alignment algorithm from MEGA 7.0 software (http://www.megasoftware.net/), and bootstrap values of 1000 were used. Amino acid sequences were deduced from 3′-terminal conserved region of ORF1b gene segments and constructed phylogenetic tree (Fig. 1). Phylogenetic analysis of selected CAstV sequences revealed that there were three lineages (Group 1, 2 and 3). In the study, 39 isolates were characterized as the member of the genus *Mamastrovirus,* but displayed wide genetic diversity, shared 77.7–100% amino acid identity with each other. Among these CAstVs, 16 isolates were clustered into *Mamastrovirus* 5, which were described previously and more closely related to CAstVs, sharing with the identity of 76.0–100% at the amino acid level, compared with CAstVs originated from Italian and UK (Group 2). Notably, there was a small branch (GX914, GX74, GX771, GXK84 and GXL435), only sharing 76.0–86.8% amino acid identity with other CAstV lineage (Group 2a). It was found that these isolates have 16 amino acids changed in the C-terminal of ORF1b gene (Fig. 2), suggesting that it is a novel CAstV lineage. Importantly, except for the group of *Mamastrovirus* 5/CAstV, there was higher similarity with MAstV3 for PoAstV5 (87.6–98.3% amino acid identity) and PoAstV2 (84.3–100% amino acid identity), which belonged to novel genotypes circulating in pig population in China [20, 21]. This is the first report that showed CAstVs share higher homology with other lineage, suggesting that there may be potential recombination events in CAstVs infection. As previously reported, the ORF1b/ORF2 region was known as a crossover point [22–24]. There were several appearance of recombination event occurred in this region from different lineages or among same serotype [23–26]. Unfortunately, we failed to amplify the whole genome of CAstVs, as it was hard to evaluate the actual origin or recombination with other species.

**Fig. 1:**
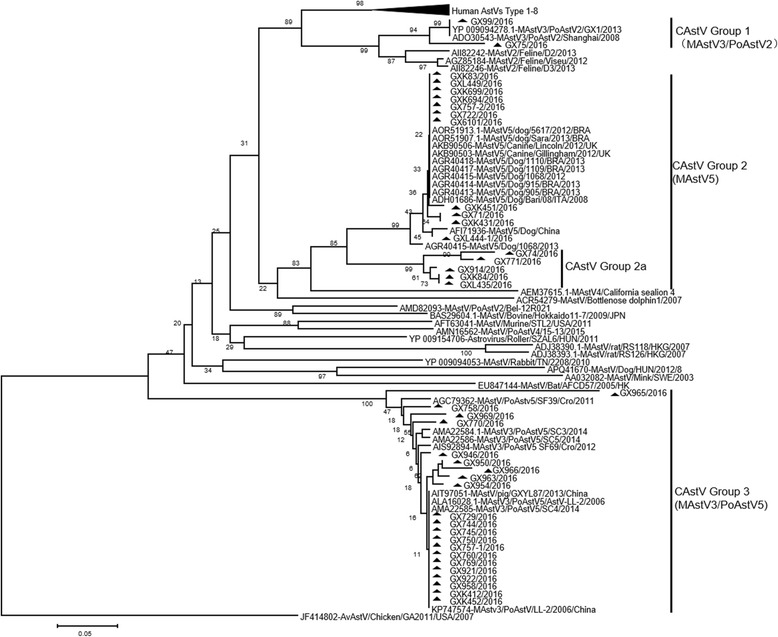
Phylogenetic tree of the C-terminal amino acid sequences (117aa) of RNA-dependent RNA polymerase (RdRp gene). The trees were generated with the MEGA 7.0 program by using neighbor-joining method with the p-distance correction and branching order reliability was evaluated by 1000 replications of a bootstrap. Triangle indicates viruses were detected in our study

**Fig. 2:**
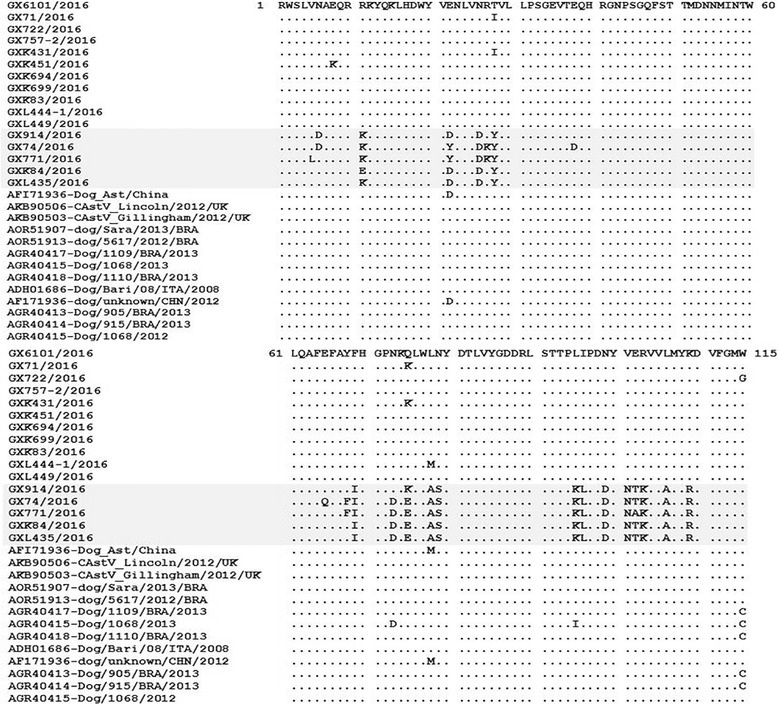
Alignment of the 16 deduced amino acid sequences of the C-terminal 115 amino acids of the RdRp of Group 1 CAstVs strains. Grey region indicates the responding Group 2a in the study

With the lack of sufficient rectal sample, only 13 partial ORF2 sequences of 79 isolates were successful to be amplified with specific primers (501F-5’CTAACAATC GTGGTCGCAAG, 1156R-5’TTGATTTGTGCATCCTTGTCC) targeting the conserved capsid gene and amplified a 634 bp fragment [18]. Comparing with all of partial ORF2 genes from China, Italy, UK, Brazil, and French, it was suggested that CAstVs may exist different genotype like porcine and human Astroviruses, some of them have association with the geographic location, like China [12], Italy [10, 11], French [18], but UK isolates and some France isolates owned a high level of genetic diversity, which were distributed in different groups [17, 18] (Fig. 3). Unlike the previous China isolates, 13 capsid strains in this study shared 93.4–100% at the amino acid level and 80.9–95.2% at the nucleotide level between strains and were grouped into two branches which were closed to China-like or Italy-like sublineage. In fact, the higher rate of evolution existed in all of AstVs, due to the increase of RNA recombination. So far, there are over 80 avian and mammalian host species detected in many species, which leads to its large diversity [27]. Generally, the majority of AstVs naturally infect only related host species, or more than one lineage could transmit in a single host [23, 28]. Whether this recombination event or cross-species transmission will emerge in CAstVs should raise corncens. Currently, the genetic diversity of the CAstV strain HUN/2012/8 with higher similarity of ORF1b and ORF2 to a mink astrovirus suggested the possible occurrence of inter-species transmission in dogs, posing a challenge for understanding the biological properties associated with viral fitness or virulence [29].

**Fig. 3:**
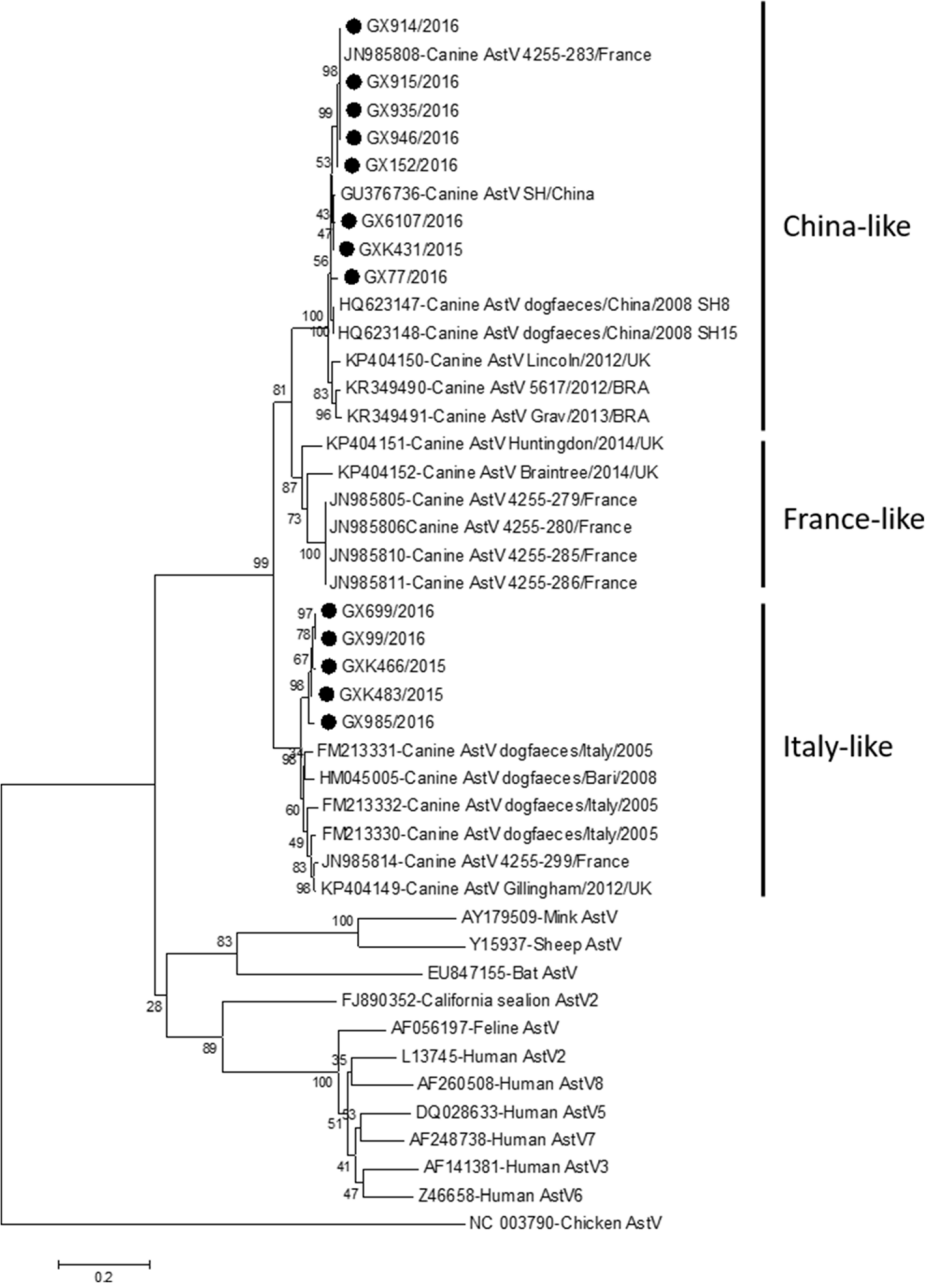
Phylogenetic tree based on partial amino acid sequences of the capsid protein (ORF2 gene) of CAstV. The solid circle indicates my isolates in this study. The trees were reconstructed using neighbor-joining method by the MEGA 6 program and Bootstrap values were based on 1000 replicates

Taken together, the study not only showed that CAstVs commonly circulate in pet dog population of Guangxi, but also found the significant genetic diversity within the CAstVs isolates. To our knowledge, this is the first report that there were two genetically diverse groups identified by ORF1b gene of CAstVs which were closely related to MAstV3, suggesting potential recombination or due to inter species transmission. Therefore, the implementation of amplification of full genome or improving CAstVs isolation will be necessary to identify the emerging recombination of CAstVs or further explore the pathogenic role of CAstVs.

The partial RNA dependent RNA polymerase sequences have been submitted to GenBank. The accession numbers were from KY271968 to KY27200). The 13 partial capsid sequences have been deposited in GenBank. The accession numbers were from KY271968 to KY272006.

## Abbreviations

AstVs: Astroviruses
CAstVs: Canine astroviruses
CCoV: Canine coronavirus
CDV: Canine distemper virus
CPV: Canine parvovirus
PoAstVs: Porcine astroviruses
RT-PCR: Reverse transcriptase-polymerase chain reaction
